# *Ecballium elaterium* attenuates neuroinflammation in an animal model of Alzheimer’s disease through modulation of nuclear factor κB pathway 

**DOI:** 10.22038/AJP.2021.18881

**Published:** 2022

**Authors:** Soomaayeh Heysieattalab, Leila Sadeghi

**Affiliations:** 1 *Department of Cognitive Neuroscience, University of Tabriz, Tabriz, Iran*; 2 *Department of Animal Biology, Faculty of Natural Sciences, University of Tabriz, Tabriz, Iran*

**Keywords:** Ecballium elaterium, Anti-inflammatory effect, NBM lesion, Oxidative stress, Cognitive dysfunction, NF-κB cascade

## Abstract

**Objective:**

Sustained inﬂammation, which could be promoted by Aβ aggregation and tau hyperphosphorylation, is a critical player in Alzheimer's disease (AD) pathogenesis. In the first phase, this study was designed to evaluate the anti-inflammatory properties of *Ecballium elaterium* (*EE*), as a Mediterranean therapeutic plant, and its effects on biochemical and behavioral signs of nucleus basalis of Meynert lesioned (NBML) rats, as an approved model of AD. In the second phase, we investigated the effect of *EE* on nuclear factor (NF)-κB pathway which is responsible for encoding proteins involved in the inflammatory cascade.

**Materials and Methods:**

Animals were divided randomly into four groups as following: control, NBML rats (AD), AD rats that were treated by high- and low-dose *EE.* Prostaglandins (PGs) levels were measured by enzyme-linked immunosorbent assay (ELISA) kits. Cyclooxygenase-2 (COX-2) and acetylcholinesterase (AChE) levels were assessed by fluorometric kit and Elman method, respectively. Behavioral signs were evaluated by Morris Water Maze (MWM) test and inflammatory proteins content was analyzed by immunoblotting method.

**Results:**

According to the results, treatment of NBML rats with *EE* fruit juice reduced PGs and cytokines more than 2-fold in comparison with AD rats through inhibition of COX-2 enzyme. Attenuation of inflammatory response in NBML rats was accompanied by reduced AChE activity (about 3-fold) and improved learning ability. Interestingly, *EE* reduced NF-κB expression for about 3-fold which resulted in a more than 10-fold increase in IκBα/P-IκBα ratio.

**Conclusion:**

Our results confirmed the TNF-α/cytokines/NF-κB/COX-2 pathway involves as the main inflammatory response in NBML rats. We also provided biochemical and behavioral evidence which introduces *EE* as an anti-inflammatory adjuvant to improve pathophysiological signs in patients suffering from AD and related dementia.

## Introduction

Alzheimer’s disease (AD) is the most common type of age-related dementia which is accompanied by neurobehavioral abnormalities that worsen over time (Karantzoulis and Galvin, 2011 ▶). AD is characterized by accumulation of extracellular amyloid plaques and intracellular neurofibrillary tangles from a neuropathological point of view (Dong et al., 2012 ▶). However, increasing evidence demonstrates a critical role for neuroinflammation cascades in AD pathology and development (Meraz-Ríos et al., 2013 ▶). It still cannot be definitively stated whether inflammation is a cause, contributor, or consequence of the disorder. But, neuroinflammation role in AD is a mainstream area of research for scientists (McManus and Heneka, 2017 ▶; Wyss-Coray and Rogers, 2012 ▶). Previous studies revealed that intraneuronal accumulation of amyloid beta (Aβ) triggers the expression of proinflammatory genes that results in increased levels of both arachidonic acid and eicosanoids derivates including prostaglandins (PGs) and cytokines (Currais et al., 2017 ▶; Welikovitch et al., 2017 ▶). It seems that proinﬂammatory factors initially have a neuroprotective role, but subsequently, can cause further neurodegeneration in a dose-dependent manner (Glass et al., 2010 ▶). Despite the ambiguous correlation between inflammation and AD, anti-inflammatory drugs have shown potential to attenuate dementia symptoms (Imbimbo et al., 2010 ▶) and inflammatory markers are critical targets for prevention, diagnosis, and treatment of AD. As we know some of herbal medicines which consist active natural compounds with anti-inflammatory properties have potential to AD treatment through impact on different neuroinflammatory molecular pathways (Akram and Nawaz, 2017 ▶). 

*Ecballium elaterium* (*EE*) is a therapeutic plant which is known as squirting cucumber and commonly grows in Mediterranean countries (Boukef, 1986 ▶; Han and Bulut, 2015 ▶). It has been used in traditional medicine for various therapeutic applications including liver cirrhosis, rheumatism, hemorrhoids and sinusitis (Attard et al., 2005; Arslan et al., 2016). Chemical composition of *EE* is dependent on growing area but it contains cucurbitacins, flavonoids, poly phenols and coumarins which are recognized to possess a wide spectrum of pharmacological properties such as anti-cancer, antimicrobial, antioxidant and anti-inflammation activities (Attard et al., 2005; Jacquot et al., 2004 ▶; Abbassi et al., 2014 ▶). Previous experiments proven that *EE*-derived cucurbitacins have neuroprotective effects in Parkinson’s disease (Arel-Dubeau et al., 2014 ▶). Although some anti-inﬂammatory properties of *EE* were shown in the neural system (Abbassi et al., 2014 ▶), there are no reports about the effects of *EE* on inflammation or following pathophysiological signs in AD patients or related animal models. As we know, lesion of the nucleus basalis of Meynert (NBM) is an approved model to study the cognitive deficit and behavioral alterations similar to AD (Kumbhare et al., 2018 ▶). So, NBM lesioned (NBML) rats have been used as an AD model in the present experiment. Thus, the aim of this study was to investigate the effects of *EE* on proinﬂammatory markers in NBML rats such as PGs (PGF2α and PGE2) and expression of interleukin 1B (IL-1B), IL-6, and tumor necrosis factor alpha (TNF-α) by mediating the nuclear factor (NF)-κB signaling in the hippocampus tissue. We also assessed neurochemical signs like oxidative stress, cholinergic function and behavioral abnormalities in experimental groups. The present study was also designed to examine the involvement of TNF-α/cytokines/NF-κB/COX-2 signaling pathway in development of AD-like symptoms in NBML rat model of AD. 

## Materials and Methods

**Chemicals**

Anti-IL-6 antibody (ab9324), Anti-IL-1β antibody (ab9787), Anti-TNFα antibody (ab6671) and Anti-NFκB antibody (ab16502) were prepared from Abcam Company. Anti-IκBα antibody (9242S), anti-phospho-IκBα antibody (9246S) and anti β-actin antibody were purchased from Cell Signaling Technology. All other solvents and chemicals were of the highest grade-commercially available. 

To prepare the fruit juice, fresh fruits of *EE *were collected in spring and thoroughly washed with tap water and fruits juice was obtained using a blender. Freshly prepared juice was filtered to remove fruit fragments before application (Celik and Aslantürk, 2009). *EE* juice was diluted in 1 ml saline freshly before administration by gavage to ensure a full dose and volume of solution was taken by the animal. 

**Experimental design**

**Figure 1:**
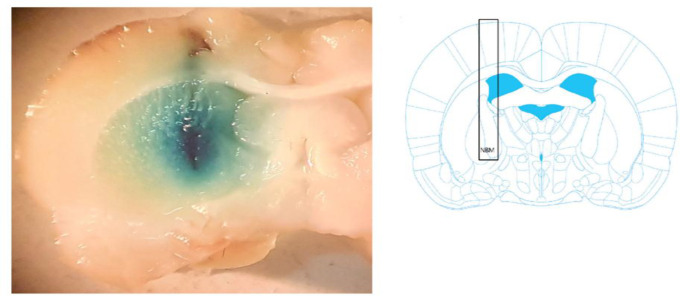
Representative brain section shows cannula placement in the NBM

Our *in vivo* study was conducted on 4.5 to 6-month-olds adult male Wistar rats weighing 250-300 g, which were obtained from Pasteur Institute of Iran. All animal works were done in University of Tabriz and approved by the Ethical Committee of Tabriz University of Medical Science (Tabriz, Iran), and they conformed to the European Communities Council Directive of 24 November 1986 (86/609/EEC). Animal experiments were carried out at 20-25°C and company prepared compressed food and municipal tap water were supplied. To prepare NBML rats as an AD model, animals were placed into a stereotaxic instrument (Stoelting, USA) after anesthetizing using an intraperitoneal injection of ketamine (125 mg/kg) and xylazine (10 mg/kg) (Sadeghi et al., 2018 ▶). The scalp of each rat was incised along the midline and bregma and lambda points were clearly visible; then, small burr holes were bilaterally drilled on the skull surface. Stereotaxic coordinates were taken from the rat brain atlas of Paxinos and Watson, 1989: Anterior–posterior (AP), 1.2 mm; medial–lateral (ML), ±3.2 mm from the central sagittal line and dorsal–ventral (DV), -7.5 mm to the surface of the skull. For bilateral injection of ibotenic acid into the NBM, 22-gauge stainless steel guide cannula were positioned and secured using dental cement (1 mm above NBM), then 1 μl of 5 μg/μl ibotenic acid solution was microinjected in each side of NBM by using microinjection pump at the speed of 120 μl/h (Figure 1). After 7 days of recovery, animals were divided randomly into four groups as following: 

1) Control: rats (n=10) were microinjected with 1 µl saline in NBM nucleus in order to simulate the equivalent changes induced by the surgery. After recovery, some of control rats (n=5) were orally administrated 1 ml saline and some of them (n=5) received 6 µl of *EE* during 25 days to evaluate the effects of fruit juice on healthy rats. 

2) NBML rats (AD): NBM nucleus of rats (n=8) was destroyed through ibotenic acid microinjection. Animals received 1 ml saline orally during 25 days after recovery. 

3) AD rats that were treated by low-dose *EE*: NBML rats (n=8) that were administered daily (oral) by 3 µl *EE* juice for 25 days post-surgery recovery. 

4) AD rats that received high-dose *EE*: NBML rats (n=8) that were treated daily with 6 µl of *EE* for 25 days after recovery.

**Measurement of PGE2 and PGF2α levels**

PGE2 and PGF2α levels were measured by the enzyme-linked immunosorbent assay (ELISA) kits (Neogen Corporation) according to the manual.

**Hippocampal COX-2 enzyme activity assay**

COX-2 catalytic activity was evaluated in hippocampus tissue homogenate by using a fluorometric kit prepared from Abcam Company, UK (ab204699) in the presence of COX-1 inhibitor according to the manual.

**Hippocampal acetylcholinesterase (AChE) activity measurement**

According to our previous work, Elman method was used to measure AChE activity in crude extract of experimental rat hippocampus (Heysieattalab and Sadeghi 2020 ▶).

**Behavioral assessment by Morris water maze (MWM)**

MWM is a rodents’ spatial learning test which evaluates navigation from start location around the perimeter of an open swimming field to an escape platform according to distal marks (Bromley-Brits et al., 2011 ▶). The water maze apparatus consists of a black-painted circular pool (155 cm diameter and 60 cm height) that is ﬁlled to a depth of 35 cm with water (22±1℃). A hidden platform was located 2 cm under the water surface in the center of the eastern quadrant. Before testing, all the rats were trained for 60 sec in the absence of the platform. Next, rats were submitted for 5 tests on three consecutive days. During the test, each rat was located in the water at the same point in the pool and allowed to swim to reach the platform and escape from the water. The rat was allowed to remain on the platform for 30 sec before the next test. Rats were dried, kept warm and returned to their home cage after each test. Swimming speed was evaluated to assess the motor activity of the rats. Duration of stay in target quadrant in the probe test was used to assess how the rats remembered the location of the platform. Visual acuity of rats was also tested by visible platform.

**Western blot analysis**

Some of the important signs of AD pathophysiology are proinﬂammatory factors accumulation, cholinergic deficits and following Aβ deposition (Dong et al., 2012 ▶). Therefore, this study evaluated NF-κB, IκBα (inhibitor of NF-κB) and phosphorylated IκBα (P-IκBα) proteins content which work together in an inflammatory signaling pathway (Oeckinghaus and Ghosh 2009 ▶). IκBα is an inhibitory protein which binds to NF-κB normally, but in inflammation status, it is phosphorylated by a specific kinase enzyme so, it could not inhibit NF-κB (Oeckinghaus and Ghosh, 2009 ▶). Related proteins content was assessed by immunoblotting method according to our previous work (Heysieattalab and Sadeghi, 2020 ▶). 

**Reactive oxygen species (ROS) measurement**

ROS generation was assessed according to our previous work by using DCFH-DA as substrate (2ʹ,7ʹ-Dichlorofluorescin Diacetate) (Heysieattalab and Sadeghi 2020 ▶).

**Statistical analysis**

Data analysis was done by Prism 6.0 (GraphPad Software Inc.) and data is presented as mean±SEM. In behavioral study, all data were analyzed using one-way ANOVA. In biochemical and immunoblotting studies, one-way ANOVA was followed by *post hoc* analysis (Tukey's test) to identify differences between the experimental groups and the control group. Differences at p<0.05 were considered significant. 

## Results

***E. elaterium*** **decreases PGs concentration in NBML rats through COX-2 enzyme regulation**

**Figure 2:**
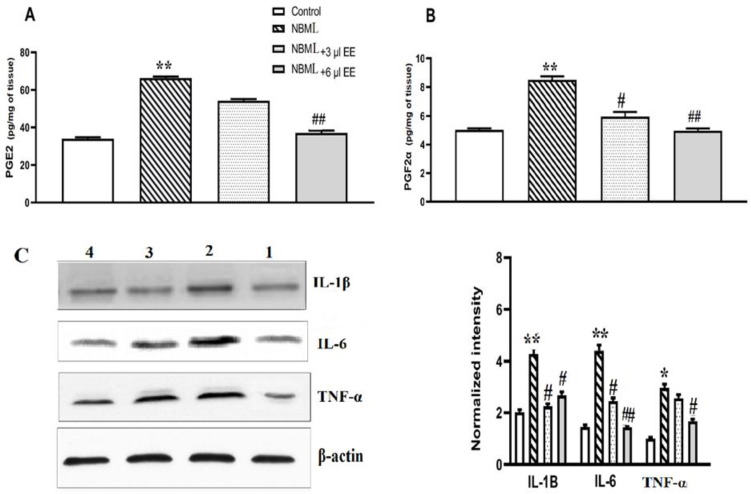
*EE* decreased PGF2α and PGE2 content of hippocampus tissue in NBML rats. High content of (A) PGF2α and (B) PGE2 was reduced in the presence of plant active compounds. (C) NBM lesion caused up-regulation of some proinflammatory proteins (IL-1B and IL-6) while *EE* fruit juice improved NBML-imposed dysregulation. Daily administration of *EE* juice could reduce both of the interleukin’s content in hippocampus tissue to near that of the control rats. Data are expressed as mean±SEM. **p<0.01, one-way ANOVA, control vs. NBM lesion. #p<0.05 and ##p<0.01, one-way ANOVA, NBM lesion vs. NBM lesion-*EE* treated rats

Our results revealed that bilateral lesions in NBM caused significant increases in PGE2 (Figure 2A, p<0.01) and PGF2α levels (Figure 2B, p<0.01) in rats hippocampus which refer to acute inflammation. However, PGE2 and PGF2α contents in the hippocampus of NBML rats that received high-dose *EE* significantly decreased as compared to the AD group (Figures 2A and 2B, p<0.01). Interestingly, low-dose *EE* could only reduce the level of PGF2α significantly (Figure 2B, p<0.05) but not PGE2 in comparison to the AD rats (p>0.05).

To further characterize the role of *EE *in anti-inflammatory events, we studied the expression of important proinflammatory proteins. According to Figure 2C, NBM lesion caused up-regulation of some proinflammatory proteins (p<0.01) while *EE* fruit juice improved NBML-imposed dysregulation. Daily administration of *EE* juice could reduce both of the interleukin’s content in hippocampus tissue to near that of the control rats. Expression of TNF-αprotein also increased significantly after NBM lesion (p<0.05). Whereas low-dose *EE* could not influence TNF-αexpression (p>0.05), high-dose treatment could reduce TNF-αcontent significantly (p<0.05). Therefore, high-dose EE reduced all of the proinflammatory proteins significantly (IL-1β: p<0.05; IL-6: p<0.01; and TNF-α p<0.05**) **in NBML rats. However, fruit juice potential to decline IL-1β and IL-6 levels was more remarkable. 

**Figure 3:**
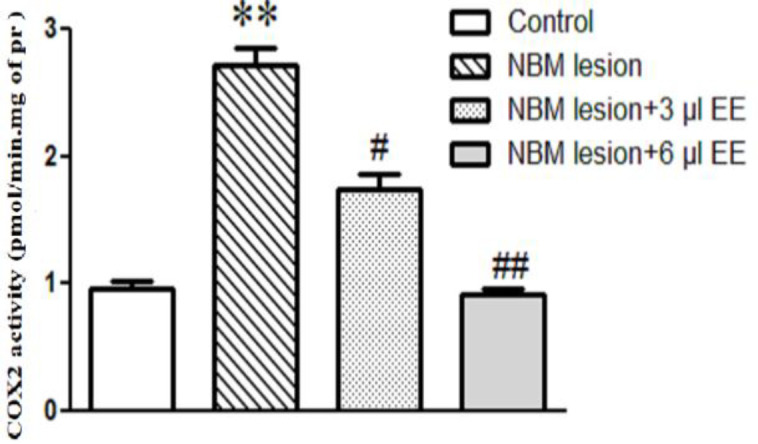
*EE* moderated COX-2 enzyme activity. Enzymatic activity of COX-2 was increased in the NBML rats while treatment of AD rats by EE juice reduced COX-2 enzyme activity to near that of the control. Each bar indicates the mean±SEM. **p<0.01, one-way ANOVA, control vs. NBM lesion. #p<0.05 and ##p<0.01, one-way ANOVA, NBM lesion vs. NBM lesion-EE treated rats

COX-2 is the main player in PGs synthesis and following inflammation which increased more than 3.5-fold in NBML group (Figure 3, p<0.01). Enzymatic activity of COX-2 was 0.96±0.067 and 2.722±0.140 pmol/min.mg protein in the control and NBML hippocampus, respectively. In 3 µl *EE*-treated NBML rats COX-2 activity showed a significant reduction (Figure 3, 1.744±0.111 pmol/min.mg protein; p<0.05). The effects of fruit juice on COX-2 activity were shown and administration of 6 µl *EE* decreased COX-2 activity to 0.932±0.045 pmol/min.mg protein (Figure 3, near that of the control; p<0.01). According to the results, *EE* could not affect none of the PG’s content or COX-2 activity significantly in the control rats (data not shown).

***EE*** **improves cholinergic system function through AChE regulation**

**Figure 4.**
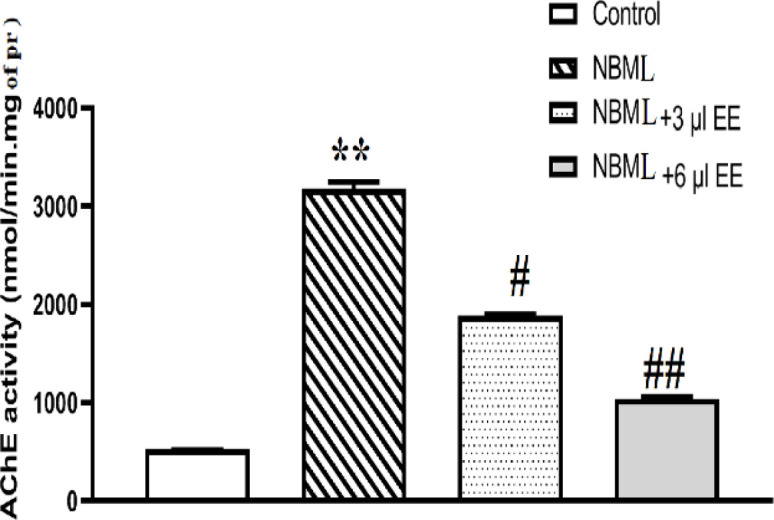
*EE* regulated AChE hydrolytic activity. NBM lesion-imposed activation of AChE referred to cholinergic dysfunction. *EE* administration reduced hippocampal AChE activity significantly in the AD rats which received low or high doses. Each bar indicates the mean±SEM. **p<0.01, one-way ANOVA, control vs. NBM lesion. #p<0.05 and ##p<0.01, one-way ANOVA, NBM lesion vs. NBM lesion-*EE* treated rats

Increased activity of AChE enzyme is one of the biological markers of AD which is associated with cholinergic deficiency (Sadeghi et al., 2018). Catalytic activity of AChE in the control was calculated to be 529.01±9.30 nmol/min.mg protein. Our results revealed that NBM lesion increased acetylcholine hydrolyzing activity in hippocampus homogenate in comparison with the controls (Figure 4, p<0.01). The obtained results showed that AChE activity significantly reduced in the NBML rats that were treated by 3 µl *EE* (Figure 4, p<0.05), enzyme activity was 3177.34±68.28 nmol/min.mg protein in the hippocampus of the NBML rats while 3 µl *EE*-treated rats showed 1883.39±19.51 nmol/min.mg protein. The NBML rats that were treated by 6 µl of *EE* showed more than 3-fold reduction in AChE activity (1031.73±27.75 nmol/min.mg protein, Figure 4, p<0.01). Therefore, *EE* reduced acetylcholine hydrolyzing activity in hippocampus tissue remarkably. Interestingly, *EE* treatment could not influence AChE activity in the healthy rats significantly (data not shown).

***EE*** **oral administration improves spatial learning ability in the NBML rats**

**Figure 5:**
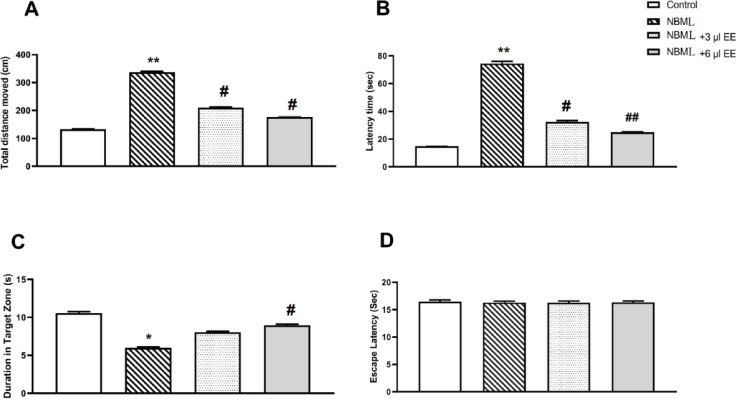
*EE* enhanced spatial learning ability in the NBML rats. In acquisition phase, distance traveled (A) and escape latency (sec) (B) to find hidden platform increased in the NBML group in comparison with the controls in 3 consecutive test days. Here, 3 and 6 µl of *EE* (n=8) significantly decreased the mean values of escape latency (sec) (A) and distance traveled to find the hidden platform (cm) (B). In probe test (C), increases in time spent in the target quadrant were observed. (D), Escape latency during visual discrimination task. Animals in different treatment conditions showed no difference in escape latency to find a visible platform. All data are expressed as mean±SEM. *p<0.05 and **p<0.01, one-way ANOVA, NBM lesion vs. Control; #p<0.05 and ##p<0.01, One-way ANOVA, NBM lesion vs. NBM lesion-*EE* treated rats

All animals learned to find the hidden platform after a three-day training. Control rats rapidly learned to swim directly to the target platform and *EE* treatment had no significant effects in control rats. AD rats needed more time explore and swimming distance increased in the NBML group as compared to the controls (Figure 5A, p<0.01). Swimming speed did not reveal any remarkable changes during the training period. In addition, the NBML rats that received saline for 25 days spent significantly more time to reach the platform as compared to the control rats (Figure 5B, p<0.01).

The NBML rats which were treated by *EE* revealed significant decreases in escape latency to reach the hidden platform (Figure 5B, 3 µl of *EE*, p<0.05; and 6 µl of *EE*, p<0.01). As shown by Figure 5A, treatment of the NBML rats with 3 and 6 µl of *EE* reduced swimming distance remarkably (3 µl of EE, p<0.05; and 6 µl of *EE*, p<0.01). There was significant difference between NBML rats which received 3 µl or 6 µl of *EE* on learning ability. Furthermore, in probe test, the NBML rats that were treated with 6 µl of *EE* spent more time in the target quadrant zone (Figure 5C, p<0.05).

Following the probe test, an additional session with a visible platform was carried out to evaluate visual perception of rats. There were no statistically significant differences in the swimming speed between groups (data not shown), therefore longer escape latency of the NBML rats was due to impaired learning instead of locomotor defects. By considering similar escape latencies to reach the visible platform (Figure 5D, p>0.05), we can conclude that there was no visual impairment in the experimental rats.

***EE*** **down-regulates proinflammatory proteins through nuclear factor (NF)-κB triggered inflammatory cascade in NBML rats’ hippocampus**

**Figure 6:**
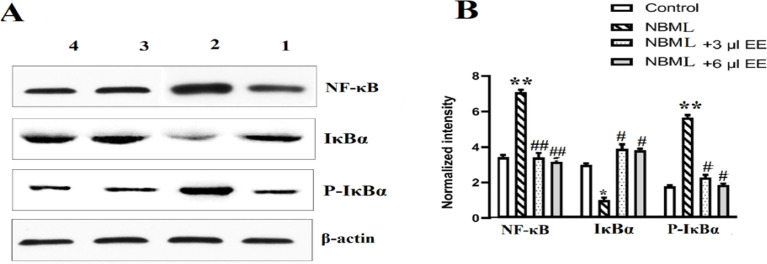
*EE* changed expression pattern of proinflammatory proteins. Western blotting analysis revealed that higher expression of cytokines in AD rats that was reduced by *EE* treatment significantly. NF-κB transcription factor, P-IκBα and IκBα expression was changed by *EE* treatment. (A) Lane 1 is related to control hippocampus, lane 2 shows results in the AD rats. Lane 3 is related to the NBML rats treated with 3 µl EE, lane 4 is related to the AD rats treated with 6 µl EE. (B) Immunoblotting results were quantified by Image J software as a plot. Each bar indicates the mean±SEM. *p<0.05 and **p<0.01, one-way ANOVA, control vs. NBM lesion. #p<0.05 and ##p<0.01, one-way ANOVA, NBM lesion vs. NBM lesion-*EE* treated rats

To clarify the functional mechanism of *EE *to improve NBML-induced PGs and cytokines overproduction, the effects of fruit juice on degradation and phosphorylation of IκBα and expression of NF-κB, were examined. Western blotting results revealed the NF-κB content increased in the NBML rats significantly rather than the control group (p<0.01). As shown in Figure 6, daily administration of 6 and 3 µl fruit juice diminished NF-κB protein amount to near that of the control group after 25 days (p<0.01). 

Our results showed that expression of NF-κB inhibitor (IκBα) was reduced in the AD group (p<0.05) while IκBα protein was mainly in phosphorylated form (p<0.05). Interestingly, administration of *EE* juice increased active form of IκBα (p<0.05) but reduced inactive form of the protein (P-IκBα) remarkably (p<0.05). It seems that total content of IκBα protein did not change in the AD group and in *EE*-received rats in comparison with control. Figure 6 shows that administration of both doses has similar results in raising IκBα protein (near to 4-fold) and also reduction of P-IκBα content (about 3-fold). 

***EE*** **decreases oxidative stress in the NBML rats**

**Figure 7:**
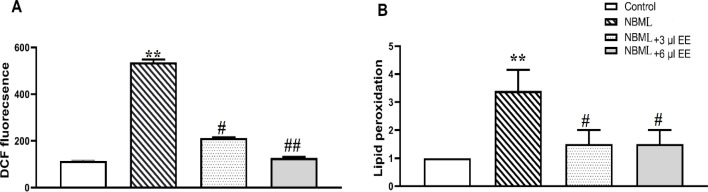
*EE* moderate the NBML induced oxidative stress. (A) DCF fluorescence indicates ROS content in hippocampus tissue related to experimental groups. (B) Lipid peroxidation in the NBML rats was increased significantly but reduced by *EE* treatment. Each bar indicates the mean ± SEM. **p<0.01, one-way ANOVA, control vs. NBM lesion. #p<0.05 and ##p<0.01, one-way ANOVA, NBM lesion vs. NBM lesion-*EE* treated rats

According to Figure 7A, rising of the DCF fluorescence in the hippocampus of the NBML rats refers to ROS overproduction in the AD model (p<0.001) which significantly reduced by EE treatment (3 µl of EE, p<0.05 and 6 µl of *EE*, p<0.01). Increase of MDA reveled cell membrane injuries in the NBML rats (p<0.001) which were improved by EE administration (Figure 7B, p<0.05).

## Discussion

Growing evidence confirms that neuroinflammation plays a critical role in initiation and development of Alzheimer’s symptoms (Meraz-Ríos et al., 2013 ▶; Wyss- Coray and Rogers, 2012 ▶). Therefore, non-steroidal anti-inflammatory drugs (NSAIDs), which work mainly through COX inhibition, are useful in AD symptoms relief (Imbimbo et al., 2010 ▶). Our literature review revealed that medicinal plants and natural compounds with anti-inflammatory features slow the progression or cause a delay in the onset of AD pathophysiological signs (Akram and Nawaz, 2017 ▶). Our primary experiments revealed that bilateral lesion of NBM causes acute inflammation in hippocampus tissue which accompanied by overproduction of PGs (PGE2 and PGF2α) and hyperactivation of COX-2 enzyme (Figures 2 and 3). Therefore, this study aimed to investigate the participation of TNF-α/cytokines/NF-κB/COX-2 signaling pathway in an NBML rat model of AD. This is also the first report the effects of *EE*, an antioxidant and anti-inflammatory plant, on crosstalk between neuroinflammation cascade and pathophysiological signs of AD like cholinergic deficits and cognitive abnormalities in NBML rat model. 

**Figure 8:**
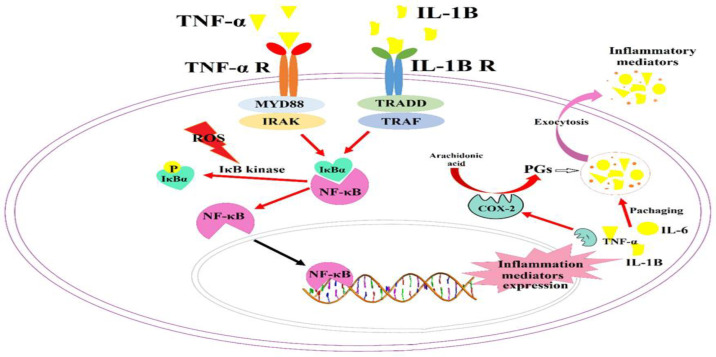
Schematic illustration of inflammation signaling pathway in AD. Inflammatory cascade which was triggered by NBM lesion primarily involved NF-κB which regulates cytokines and COX-2 expression. IκBα/P-IκBα ratio, which was regulated by ROS molecules, directly amplifies the cascade. *EE* fruit juice could remarkably attenuate NF-κB pathway so, inhibit inflammation and following events

Our results showed that NBM lesion triggers an increase in inflammatory cytokines content such as IL-6, TNF-α and IL-1β in hippocampus tissue (Figure 3), that is in agreement with a previous study (Meraz-Ríos et al., 2013 ▶). Figure 8 schematically illustrates the investigated molecular pathway in details. Increased interleukins and TNF-α convert NF-κB/IκBα complex to the active form (Oeckinghaus and Ghosh, 2009 ▶) which increases cytokines and chemokines expression, such as interferons, interleukins, lymphokines and TNF-α, so, amplify the inflammation process during positive feedback (Liu et al., 2017 ▶). Our results confirmed that NF-κB and its positive regulator (P-IκBα) increased as a consequence of NBM lesion which refer to neuroinflammation (Liu et al., 2017 ▶). Figure 6 revealed that NBML reduced IκBα less than one third and increased P-IκBα about 4-fold which could be refer to NF-κB activation in AD rats. Therefore, inflammation has a direct relationship with IκBα/P-IκBα ratio which could also be promoted by oxidative stress (Figure 7). Final result of NF-κB activation is up-regulation of inflammatory mediators such as IL-6, IL-1β, TNF-α and COX-2 (Ricciotti and FitzGerald 2011 ▶) which was also approved by our results (Figures 2 and 3). 

According to previous results, *EE* contains different active compounds such as flavonoids, cucurbitacins and polyphenols (Felhi et al., 2016 ▶; Bourebaba et al., 2020 ▶) which could affect the NBM lesion-imposed inflammation primarily through decreasing NF-κB content in hippocampus tissue which down-regulates cytokines such as IL-6, IL-1β and TNF-α during a negative regulation (Oeckinghaus and Ghosh 2009 ▶). Cucurbitacins have neuroprotective roles and decrease neurodegeneration in Parkinson's disease (Arel-Dubeau et al., 2014 ▶). As our results showed, *EE* administration causes reduction of IκBα/P-IκBα ratio near to one tenth through direct interaction with the related kinase enzyme or indirectly by ROS content reduction (Morgan and Liu 2011 ▶). Analysis of *EE* proved that it has a couple of natural products with free OH groups which could attach to the ROS molecules and neutralize them (Felhi et al., 2016 ▶; Bourebaba et al., 2020 ▶) so reducing ROS content near to the one fifth (Figure 7). Consequently, low-dose *EE* could reduce evaluated cytokines near to the half and high-dose *EE* decreased it more remarkably (3-fold). 

In agreement with this study, it has been shown that COX-2 is up-regulated in AD patients (Ho et al., 2001 ▶). Based on our results, *EE* could inhibit COX-2 activity possibly through NF-κB pathway (Figure 3 and 8) and other cascades which need further investigation. Figure 2 shows that both of the evaluated PGs increased in the NBML rats’ hippocampus but 25-day treatment of rats with *EE* juice could reduce PGs and COX-2 activity significantly. Plant juice also inhibited hydrolytic activity of AChE enzyme significantly at both doses and improved cholinergic deficiency (Figure 4). It’s possible that active compounds in *EE* could regulate AChE directly or indirectly by modulating signaling pathways that needs further investigation. 

In addition to cytokines and oxidative stress, other molecular events such as amyloid plaques deposition could also promote NF-κB signaling pathway by phosphorylation of IκBα (Currais et al., 2017 ▶; Morgan and Liu, 2011 ▶). This cycle (inflammation/oxidative stress/amyloid deposition) promotes neurodegeneration and cognitive impairments which were assessed by MWM test. The rats which suffered from NBM lesion and showed high concentration of cytokines and PGs and also activation of NF-κB signaling also spent more time to reach the platform. Interestingly, attenuation of TNF-α/cytokines/NF-κB/COX-2 pathway by plant juice treatment significantly reduced cholinergic deficits and also improved hippocampal-dependent learning ability (Figure 8). 

Taken together, our results revealed a positive feedback-loop between neuroinflammation, cholinergic deficits, and neurochemical and behavioral abnormality in AD. This loop seems as an effective risk factor in initiation and development of the AD and related neurodegenerative disorders. It is strongly suggested that local administration of ibotenic acid promotes inﬂammation-induced memory impairment by mediation of oxidative stress, cytokines and cholinergic dysfunction. While *EE* could reduce pathophysiological symptoms related to AD through inhibition of inflammatory pathway (TNF-α/cytokines/NF-κB/COX-2) and also cholinergic system. By considering phytochemical composition of *EE* which consists of different antioxidants such as ferulic acid, kaempferol, and diosmetin and anti-inflammatory chemicals such as cucurbitacin D, E, and P and other flavonoids (Felhi et al., 2016 ▶; Bourebaba et al., 2020 ▶), it could be used as a therapeutic adjuvant in patients suffering from AD or related cognitive disorders.

## Conflicts of interest

The authors have declared that there is no conflict of interest.

## References

[B1] Abbassi F, Ayari B, Mhamdi B, Toumi L (2014). Phenolic contents and antimicrobial activity of squirting cucumber (Ecballium elaterium) extracts against food-borne pathogens. Pak J Pharm Sci.

[B2] Akram M, Nawaz A (2017). Effects of medicinal plants on Alzheimer's disease and memory deficits. Neural Regen Res.

[B3] Arel-Dubeau A, Longprè F, Bournival J, Tremblay C, Demers-Lamarche J, Haskova P (2014). Cucurbitacin E has neuroprotective properties and autophagic modulating activities on dopaminergic neurons. Oxid Med Cell Longev.

[B4] Arslan MS, Basuguy E, Ibiloglu I, Bozdemir E, Zeytun H, Sahin A, Kaplan I, Aydogdu B, Otcu S (2016). Effects of Ecballium elaterium on proinflammatory cytokines in a rat model of sepsis. J Invest Surg.

[B5] Boukef K (1986). Les Plates Dans la Me´ decine Traditionnelle Tunisienne.

[B6] Bourebaba L, Gilbert-Lopez B, Oukil N, Bedjou F (2020). Phytochemical composition of Ecballium elaterium extracts with antioxidant and anti-inflammatory activities: Comparison among leaves, flowers and fruits extracts. Arab J Chem.

[B7] Bourebaba L, Gilbert-López B, Oukil N, Bedjou F (2020). Phytochemical composition of Ecballium elaterium extracts with antioxidant and anti-inflammatory activities: Comparison among leaves, flowers and fruits extracts. Arab J Chem.

[B8] Bradford MM (1976). Rapid and sensitive method for the quantitation of microgram quantities of protein utilizing the principle of protein-dye binding. Anal Biochem.

[B9] Buege JA, Aust SD, Flesicher, S, Packer, L (1978). Microsomal lipid, Peroxidation.

[B10] Bromley-Brits K, Deng Y, Song W (2011). Morris water maze test for learning and memory deficits in Alzheimer's disease model mice. J Vis Exp.

[B11] Celik TA, Aslantürk OS (2009). Investigation of cytotoxic and genotoxic effects of Ecballium elaterium juice based on Allium test. Methods Find Exp Clin Pharmacol.

[B12] Currais A, Fischer W, Maher P, Schubert D (2017). Intraneuronal protein aggregation as a trigger for inflammation and neurodegeneration in the aging brain. FASEB J.

[B13] Dong S, Duan Y, Hu Y, Zhao Z (2012). Advances in the pathogenesis of Alzheimer's disease a re-evaluation of amyloid cascadehypothesis. Transl Neurodegener.

[B14] Felhi S, Hajlaoui H, Ncir M, Bakari S, Ktari N, Saoudi M, Gharsallah N, Kadri A (2016). Nutritional, phytochemical and antioxidant evaluation and FT-IR analysis of freeze dried extracts of Ecballium elaterium fruit juice from three localities. Food Sci Technol.

[B15] Glass CK, Saijo K, Winner B, Marchetto MC, Gage FH (2010). Mechanisms underlying inflammation in neurodegeneration. Cell.

[B16] Han MI, Bulut G (2015). The folk-medicinal plants of Kadis ehri (Yozgat-Turkey). Acta Soc Bot Pol.

[B17] Heysieattalab S, Sadeghi L (2020). Effects of delphinidin on pathophysiological signs of nucleus basalis of meynert lesioned rats as animal model of Alzheimer disease. Neurochem Res.

[B18] Ho L, Purohit D, Haroutunian V, Luterman JD, Willis F, Naslund J (2001). Neuronal cyclooxygenase 2 expression in the hippocampal formation as a function of the clinical progression of Alzheimer disease. Arch Neurol.

[B19] Imbimbo BP, Solfrizzi V, Panza F (2010). Are NSAIDs useful to treat Alzheimer's disease or mild cognitive impairment?. Front Aging Neurosci.

[B20] Jacquot C, Rousseau B, Carbonelle D, Chinou I, Malleter M, Tomasoni C, Roussakis C (2014). Cucurbitacin-D-induced CDK1 mRNA up-regulation causes proliferation arrest of among-small cell lung carcinoma cell line (NSCLC-N6). Anticancer Res.

[B21] Karantzoulis S, Galvin JE (2011). Distinguishing Alzheimer's disease from other major forms of dementia. Expert Rev Neurother.

[B22] Kumbhare D, Palys V, Toms J, Wickramasinghe CS, Amarasinghe K, Manic M, Hughes E, Holloway K (2018). Nucleus basalis of meynert stimulation for dementia: Theoretical and technical considerations. Front Neurosci.

[B23] Liu T, Zhang L, Joo D, Sun SC (2017). NF-κB signaling in inflammation. Signal Transd Targ Therap.

[B24] McManus RM, Heneka MT (2017). Role of neuroinflammation in neurodegeneration: new insight. Alzheimers Res Ther.

[B25] Meraz-Ríos MA, Toral-Rios D, Franco-Bocanegra D, Villeda-Hernández J, Campos-Peña V (2013). Inflammatory process in Alzheimer's disease. Front Integr Neurosci.

[B26] Morgan MJ, Liu ZG (2011). Crosstalk of reactive oxygen species and NF-κB signaling. Cell Res.

[B27] Oeckinghaus A, Ghosh S (2009). The NF-kappaB family of transcription factors and its regulation. Cold Spring Harb Perspect Biol.

[B28] Paxinos G, Watson C (1986). The Rat Brain in Stereotaxic Coordinates.

[B29] Sadeghi L, Yousefi Babadi V, Tanwir F (2018a). Improving effects of Echium amoenum aqueous extract on rat model of Alzheimer's disease. J Integr Neurosci.

[B30] Sadeghi L, Tanwir F, Yousefi Babadi V (2018). Physiological and biochemical effects of Echium amoenum extract on Mn2+-imposed Parkinson like disorder in rats. Adv Pharm Bull.

[B31] Sarnyai Z, Sibille EL, Pavlides C, Fenster RJ, McEwen BS, Toth M (2000). Impaired hippocampal-dependent learning and functional abnormalities in the hippocampus in mice lacking serotonin1A receptors. Proc Natl Acad Sci USA.

[B32] Ricciotti E, FitzGerald GA (2011). Prostaglandins and inflammation. Arterioscler Thromb Vasc Biol.

[B33] Welikovitch LA, Do Carmo S, Maglóczky Z, Malcolm JC, Lőke J, Klein WL, Freund T, Cuello AC (2020). Early intraneuronal amyloid triggers neuron-derived inflammatory signaling in APP transgenic rats and human brain. Proc Natl Acad Sci USA.

[B34] Wyss-Coray T, Rogers J (2012). Inflammation in Alzheimer disease-a brief review of the basic science and clinical literature. Cold Spring Harb Perspect Med.

